# Identification of Differentially Expressed Gene Transcripts in Porcine Endometrium during Early Stages of Pregnancy

**DOI:** 10.3390/life10050068

**Published:** 2020-05-16

**Authors:** Mariusz Pierzchała, Dorota Pierzchała, Magdalena Ogłuszka, Ewa Poławska, Tadeusz Blicharski, Agnieszka Roszczyk, Agata Nawrocka, Pawel Urbański, Kamila Stepanow, Aleksandra Ciepłoch, Agnieszka Korwin-Kossakowska, Marinus F.W. te Pas, Brygida Slaska, Magdalena Buszewska-Forajta, Jedrzej M. Jaśkowski, Mateusz Sachajko, Magdalena Herudzińska, Bartosz M. Jaśkowski, Wojciech Niżański, Leyland Fraser, Urszula Czarnik, Haja N. Kadarmideen, Chandra S. Pareek

**Affiliations:** 1 Department of Animal Genomics and Biodiversity, Institute of Genetics and Animal Breeding, Polish Academy of Sciences, Jastrzebiec, 05-552 Magdalenka, Poland; mariusz.pierzchala@gmail.com (M.P.); pierzchalada@gmail.com (D.P.); m.ogluszka@ighz.pl (M.O.); ewchubby@poczta.onet.pl (E.P.); t.blicharski@ighz.pl (T.B.); a.roszczyk@ighz.pl (A.R.); a.nawrocka@ighz.pl (A.N.); p.urbanski@ighz.pl (P.U.); k.stepanow@ighz.pl (K.S.); a.cieploch@ighz.pl (A.C.); a.kossakowska@ighz.pl (A.K.-K.); 2 Wageningen UR Livestock Research, Animal Breeding and Genomics, 6708 PB Wageningen, The Netherlands; marinus.tepas@wur.nl; 3 Institute of Biological Bases of Animal Production, Faculty of Biology, Animal Sciences and Bioeconomy, University of Life Sciences in Lublin, 20-950 Lublin, Poland; brygida.slaska@up.lublin.pl; 4 Department of Biopharmaceutics and Pharmacodynamics, Faculty of Pharmacy, Medical University of Gdańsk, 80-416 Gdańsk, Poland; magdalena.buszewska-forajta@gumed.edu.pl; 5 Institute of Veterinary Medicine, Faculty of Biological and Veterinary Sciences, Nicolaus Copernicus University, 87-100 Toruń, Poland; jmjaskowski@umk.pl (J.M.J.); mateuszsachajko@gmail.com (M.S.); mherudzinska@umk.pl (M.H.); 6 Department of Reproduction and Clinic of Farm Animals, Faculty of Veterinary Medicine, Wrocław University of Environmental and Life Science, 50-366 Wrocław, Poland; bmjaskowski@gmail.com (B.M.J.); wojciech.nizanski@upwr.edu.pl (W.N.); 7 Department of Biochemistry and Animal Biotechnology, Faculty of Animal Bio-engineering, University of Warmia and Mazury in Olsztyn, 10-719 Olsztyn, Poland; fraser@uwm.edu.pl; 8 Department of Pig Breeding, Faculty of Animal Bio-engineering, University of Warmia and Mazury in Olsztyn, 10-719 Olsztyn, Poland; czar@uwm.edu.pl; 9 Quantitative Genomics, Bioinformatics and Computational Biology Group, Department of Applied Mathematics and Computer Science, Technical University of Denmark, 2800 Kongens Lyngby, Denmark; hajak@dtu.dk; 10 Centre for Modern Interdisciplinary Technologies, Nicolaus Copernicus University, 87-100 Toruń, Poland

**Keywords:** upregulated, downregulated, differentially expressed genes, transcripts, fold change, preimplantation, embryo, pregnancy, microarray, porcine, endometrium

## Abstract

During the early stages of pregnancy, the uterine endometrium undergoes dramatic morphologic and functional changes accompanied with dynamic variation in gene expression. Pregnancy-stage specific differentially expressed gene (DEG)-transcript-probes were investigated and identified by comparing endometrium transcriptome at 9th day (9D), 12th day (12D) and 16th day (16D) of early pregnancy in Polish large-white (PLW) gilts. Endometrium comparisons between 9D-vs-12D, 9D-vs-16D and 12D-vs-16D of early pregnancy identified 6049, 374 and 6034 highly significant DEG-transcript-probes (*p* < 0.001; >2 FC). GO term enrichment analysis identified commonly shared upregulated endometrial DEG-transcript-probes (*p* < 0.001; >2 FC), that were regulating the gene functions of anatomic structure development and transport (*TG*), DNA-binding and *methyltransferase* activity (*ZBTB2*), ion-binding and *kinase* activity (*CKM*), cell proliferation and apoptosis activity (*IL1B)*. Downregulated DEG-transcript-probes (*p* < 0.001; >2 FC) were involved in regulating the gene functions of *phosphatase* activity (*PTPN11*), TC616413 gene-transcript and *Sus-scrofa* LOC100525539. Moreover, blastn comparison of microarray-probes sequences against *sus-scrofa11* assembly identified commonly shared upregulated endometrial DEG-transcript-probes (E < 0.06; >2 FC), that were regulating the gene functions of reproduction and growth (*SELENOP*), cytoskeleton organization and *kinase* activity (*CDC42BPA*), *phosphatase* activity (*MINPP1*), enzyme-binding and cell-population proliferation (VAV3), cancer-susceptibility candidate gene (*CASC4*), cytoskeletal protein-binding (*COBLL1*), ion-binding, enzyme regulator activity (*ACAP2*) Downregulated endometrial DEG-transcript-probes (E < 0.06; >2FC) were involved in regulating the gene functions of signal-transduction (*TMEM33),* catabolic and metabolic processes (*KLHL15)*. Microarray validation experiment on selected candidate genes showed complementarity to significant endometrial DEG-transcript-probes responsible for the regulation of immune response (*IL1B, S100A11*), lipid metabolism (*FABP3, PPARG*), cell-adhesion (*ITGAV*), *angiogenesis* (*IL1B*), intercellular transmission (*NMB*), cell-adhesion (*OPN*) and response to stimuli (*RBP4*) was confirmed by RT-PCR. This study provides a clue that identified pregnancy-stage specific microarray transcript probes could be considered as candidate genes for recognition and establishment of early pregnancy in the pig.

## 1. Introduction

Reproduction is an essential element of effective animal production because it has a direct impact on its profitability. The crucial point is to create an optimal environment for the maturation of reproductive cells during the estrus cycle, followed by fertilization and fetal development. During the female estrous cycle, as well as during the early pregnancy periods in the reproductive system, there are physiological changes controlled at several levels, including gene expression changes in the endometrium [1,2]. The pregnancy in pigs takes about 114 days, while implantation, like in other livestock, such as a cow or sheep, is non-invasive [3,4]. The primary role of early pregnancy is regulated by the adhesion forces that allow the embryo to adhere to the uterine walls without the need for trophoblast invasion. As a result of such implantation a placenta adeciduata, i.e., placenta spuria (placenta epitheliochorialis) and the placenta diffusa is formed [5]. In the first few weeks of pregnancy in the pig, these critical changes could be defined as the three main stages: (i) the peri-implantation period after fertilization, i.e., early pregnancy before maternal recognition of pregnancy (gestation period duration of 1–10 days: in this study 9th day), (ii) the peri-implantation period of maternal recognition of pregnancy (gestation period duration of 11–13 days: in this study 12th day) [6,7,8] and, (iii) peri-implantation period to determine establishment or failure of pregnancy (gestation period duration of 14–19 days: in this study 16th day) [9,10,11,12,13,14]. During this peri-implantation period, the embryo pre-contacts with uterine luminal epithelia followed by endometrial invasion to initiate the placentation. These events are prerequisites for fetal and placental growth and development through the remainder of pregnancy [15]. In pigs, successful embryo implantation is also essential for producing large litter size and early embryo loss occurring on day 12–30 of gestation critically affects this parameter [16]. The period around implantation is a critical moment in the course of pregnancy where the highest percentage of embryo mortality is associated with embryo defects, disorders in the normal functioning of the uterus, as well as the lack of efficient interaction of these two elements essential for implantation and functional placental attachment [17].

The present study aims to compare and identify the pregnancy-stage specific differentially expressed endometrial genes, during three different stages of early pregnancy (9D, 12D and 16D) in the Polish large-white (PLW) gilts. The PLW is one of the most commonly used commercial pig breeds in Poland, which is characterized by the high performance and reproductive traits, *viz.*, litter size, in nucleus swine herds [18]. This breed was chosen because of its high reproduction rate and usefulness as a conserved and commercial pig breed maintained in Poland [19].

## 2. Materials and Methods

### 2.1. Ethical Statement and Animal Materials

All procedures involving animals were approved by the resolution of the third local animal ethics committee no. 70/2006.

#### 2.1.1. Ethical Statement

All experiments were approved by the Animal Ethics Committee at IGHZ, Jastrzebiec, Poland and were conducted in accordance with the commission of national guidelines for agricultural animal care.

#### 2.1.2. Animal Material

In the microarray experiment, we have investigated the PLW gilts (n = 12) from the pig testing station (SKURTCh) located in Mełno, Poland. The PLW gilts weighing about 130 kg were introduced into individual pens to observe the symptoms of heat. Heat-check positive PLW gilts were bred twice to boars of the respective breed, 24 h apart, via natural service at their second or third estrus. In the established second heat, the gilts were inseminated twice, according to the methodology adopted in the mating of pigs at the intervals of 24 hours [6] and the gestation date was considered and calculated from the first day of insemination. The inseminated PLW gilts were slaughtered at 9th day (9D), 12th day (12D) and 16th day (16D) after insemination (post coitus), respectively, using IACUC approved protocols. Pregnancy was confirmed by the presence of the typical characteristic for the given embryos determined by the number of corpus luteum (Table 1). Because it was not easy to find and count embryos, we stopped searching and elution after getting up to 12 embryos from each PLW gilt on an average. Tissue collection was performed by collecting the endometrial slices from the middle part of the mesometrial side of the uterus horn (all areas of the endometrium lumen). The collected endometrium tissues were immediately frozen in liquid nitrogen and stored at −80 °C until transcriptomic analyses.

### 2.2. RNA Isolation

Total RNA was isolated from 50 mg of endometrium tissue of PLW gilts (n = 12). Samples were homogenized by MagNALyser (Roche Diagnostics Corporation, Switzerland) in guanidinium thiocyanate [20] (Qiazol) buffer (Qiagen). Homogenization was carried out in three cycles of 20 s. After each cycle, the samples were cooled at 4 °C. Total RNA was isolated using RNeasy microarray mini kit (Qiagen, Valencia, CA, USA) according to the manufacturer’s recommendations. Isolates were digested with DNase (Sigma-Aldrich Co. LLC, MO 63103, United States) according to the manufacturer’s procedure to remove the genomic DNA residues. Initial quantitative and qualitative RNA analysis was performed by spectrophotometric measurement using the NanoDrop ND-1000 apparatus (Thermo Scientific, Pittsburgh, PA, USA) and electrophoresis in 1% denaturing agarose gel with the addition of ethidium bromide. The final integrity of the isolated total RNA was verified using a BioAnalyzer 2100 instrument and Agilent 2100 Expert software (Agilent Technologies, Santa Clara, USA). The RIN (RNA integrity number) of all investigated transcriptome samples (n = 12) in the microarray experiment were above 8.

### 2.3. Laboratory Procedure of Microarray Experiment

The total RNA was transcribed into complementary cDNA using transcription first-strand cDNA synthesis kit (Roche Applied Science) according to the manufacturer’s procedure. The microarray analysis was based on the pooled all samples (n = 12) in equal proportions as common-reference sample and individual mRNA sample comparison of each of the three early pregnancy groups using Agilent two-color microarray [21]. Samples of pregnant gilts endometrium belonging to three groups of early pregnancy were referred as: (i) day 9 after insemination, i.e., post-fertilization period (n = 4); (ii) day 12 after insemination, i.e., maternal recognition of pregnancy (n = 4); and (iii) day 16 after insemination, i.e., representing the beginning of embryo implantation (n = 4). Endometrium samples were processed according to Agilent two-color microarray-based gene expression analysis using low input quick amp-labeling kit protocol, following the instructions provided by the manufacturer [22]. Finally, samples were hybridized to *sus scrofa* (Pig) V2 Gene Expression Microarray 4 × 44 microarrays (Agilent Technologies, Santa Clara, CA, USA) at 65 °C for 17 hours in a hybridization cradle oven (Agilent Technologies, CA 95051, United states).

### 2.4. Bioinformatics Analysis of Gene Expression Data Generated by Microarrays

#### 2.4.1. Processing of Microarray Raw Data Analysis

Microarrays data were scanned using an Agilent G2565CA microarray scanner system (Agilent Technologies, Santa Clara, CA, USA) and the fluorescence results were processed using feature extraction 4.0 software (Agilent Technologies, Santa Clara, CA, USA). Raw expression values were analyzed after quantile normalization using ArrayStar software (DNASTAR). Principal component analysis (PCA) [23] was conducted on endometrial microarray data (submitted to GEO NCBI resources) representing 9D, 12D and 16D of early pregnancy of PLW gilts (Table S1). Processed data were analyzed using the three levels of adjustments: (i) an unadjusted, (ii) a moderated t-test with Benjamini-Hochberg false discovery rate (FDR) determination and (iii) Bonferroni adjustment, respectively [24,25].

#### 2.4.2. Stringent Criteria of Excel-filtering of DEG-transcript-probes Microarray Data

In this study, two stringent criteria of excel filtering were applied to analyze and compare the DEG-transcript-probes at three stages (9D, 12D and 16D, respectively) of early pregnancy in PLW gilts. First, comparisons of DEG-transcript-probes among 9D, 12D and 16D, pregnancy were performed to obtain the highly significant microarray transcript probes with cutoff *p* values (cutoff *p*-values < 0.001 and fold change (FC) greater than 2 (>2 FC)). Second, comparisons of DEG-transcript-probes among 9D, 12D and 16D, pregnancy were performed to obtain the highly significant microarray transcript probes with blastn E-values and high-scoring segment pair (HSP) values of microarray probes sequences against representatives of gene-transcript of *sus scrofa* assembly *Sscrofa11.1* [26]. The results of blast alignment probe sequences to reference pig genome were presented in a table according to following parameters: lowest E-value, highest identity, highest positive%, greatest HSP length and highest bit score, respectively.

Justification of Blastn analysis of microarray data: Deep comparison of microarray probes sequences against representatives of gene-transcript of *Sus-scrofa* assembly *Sscrofa11.1* was performed using blastn by CLC-Genomics Workbench 12. Software. Obtained results (Tables S2–S10) were selected to find the best matched sequence based on lowest E-value (i.e., the number of expected hits of similar quality (score), that could be found just by chance), as well as, the higher bit-score (i.e., normalized sequence similarity normalized based on the raw pairwise alignment score) based on the highest identity and greatest high-scoring segment pair (HSP) length (BLAST^®^ help) [27].

#### 2.4.3. Visualization of Upregulated and Downregulated DEG-transcript-probes of Microarray Data using Venn Diagrams, Heatmaps and ClueGO

Three comparisons of 9D vs. 12D; 9D vs. 16D and 12D vs. 16D, respectively, were performed and the resulting expression intensity values were further visualized based on the Venn diagrams using standard protocols [28]. Additionally, heatmaps were utilized to visualize the DEGs pattern of the endometrial tissue at different stages of early pregnancy. The heatmap script was constructed from the package “gplot” as an enhanced version or its basic function stats in R [29,30]. The heatmaps were plotted from the normalized microarray data (Tables S5–S10) using heatmap function in R package (version 3.6.2) for log2-fold change. Data were scaled by rows and hierarchical computing clustering was performed based on their means. Finally, based on Tables S5–S10, the gene network and functional analysis of identified commonly sheared DEGs annotated microarray probes were visualized using ClueGO [31]. Descriptions of analyzed ClueGO parameters for all three stages of early pregnancies were presented in Table S11. Cytoscape software presented biologic role specificity of most significantly DEG-transcripts probes on the basis of their functional and pathway enrichment analyses including gene ontology database, i.e., GO_BiologicalProcess-EBI-UniProt-GOA_28.03.2020_00h00, GO_CellularComponent-EBI-UniProt-GOA_28.03.2020_00h00, GO_ImmuneSystemProcess-EBI-UniProt-GOA_28.03.2020_00h00, GO_MolecularFunction-EBI-UniProt-GOA_28.03.2020_00h00 [32].

### 2.5. Real time PCR Gene Expression Validation

For the validation of microarray results and gene expression analyses, standard method real-time PCR was used. In order to avoid the influence of additional factors on the correlation of results between the microarrays and real-time PCR, the primers were designed to include the region itself as the probe [33]. For this purpose, the Primer-BLAST software [34] was used to enable simultaneous verification of primer specificity. The quality of the primers was checked using the OligoAnalyzer 3.1 software (Integrated DNA Technologies, USA) [35]. Selected reference genes and experimentally identified DEG-transcript-probes primers sequences are presented in Table 2 and Table 3.

#### 2.5.1. Laboratory Procedure of Real-time PCR

Total RNA was transcribed into complementary cDNA using transcription first-strand cDNA synthesis kit (Roche Applied Science, Switzerland) according to the manufacturer’s procedure. Expression validation analysis was performed on a Light-Cycler 480 instrument (Roche Applied Science, Switzerland) using 5xHOT FIREPol Eva Green qPCR mix plus (no ROX) (Solis BioDyne, Estonia). The reaction mixture in a volume of 15 μL contained 140nM of each primer and 90 ng of matrices. Amplification was carried out in 42 denaturation cycles: 95 °C for 15 s, annealing 54 °C for 20 s and elongation 72 °C for 20 s, preceded by preliminary denaturation at 95 °C for 15 min. The next step was the expression analysis of melting curves for every amplicon. The determination of the quality and specificity of amplicons was based on one peak in the melting curve and the product amplification sizes verified on gel electrophoresis.

#### 2.5.2. Reference Genes Panel in the Microarray Validation Experiment

A panel of nine reference genes, namely TBP (TAT binding protein), GAPDH (*Glyceraldehyde 3-phosphate dehydrogenase*), HPRT1 (*Hypoxanthine guanine phosphoribosyltransferase 1*), BACT (β-actin), CYTB (cytochrome B), YWHAZ (*Tyrosine 3-Monooxygenase*/*Tryptophan 5-Monooxygenase* Activation Protein), B2 M (*Beta-2-microglobulin*), SDHA (*Succinate dehydrogenase complex, subunit A*), HMBSA (*Hydroxymethylbilane synthase a*) were selected to validate the microarray experiment (Table 2).

#### 2.5.3. Analysis of Reference Genes Stability

**Figure 1:**
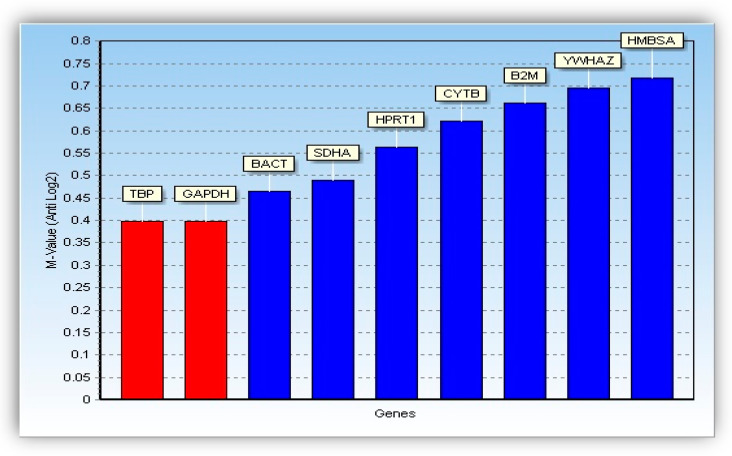
Bar diagrams showing stability measures based on M-values calculated by GeNorm.

**Figure 2:**
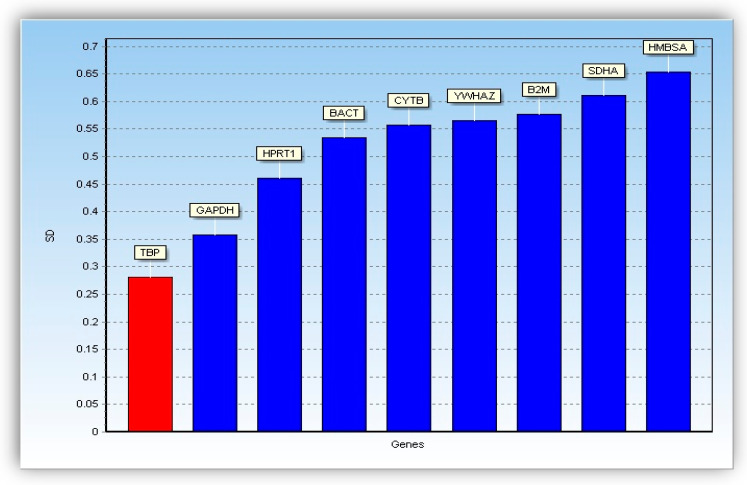
Bar diagrams showing stability measures based on standard deviations calculated by NormFinder.

Housekeeping genes used as reference genes in the analysis of gene expression by real-time PCR can be regulated under the influence of steroid hormones, hence the need to verify the stability of expression of selected genes in the endometrium. Stability of nine reference genes was verified using GenEX 5.4.4 software (MultiD Analyzes AB) of the GeNorm [36] and NormFinder [37] applications. In the case of application GeNorm, level of expression of the reference genes was compared in pairs concerning the expression of other genes. The variability of the expression of a single gene was visualized using the value of M, which is the average standard deviation for each of the gene pairs. In the case of application Norm Finder, the stability of gene expression was determined by the standard deviation (SD) of each gene. After evaluation check and stability measurements of the nine reference genes, both GeNorm (Figure 1) and Norm Finder (Figure 2) pointed-out *TBP* gene as the reference-gene with the highest expression stability, while *GAPDH* reference-gene was in second place in this respect. However, the remaining seven reference genes were found to be the least stable. Therefore, a panel of both *TBP* and *GAPDH* were finally selected as reference genes to conduct further validation analysis using RT-PCR.

#### 2.5.4. Selected DEG-transcript-probes in the Microarray Validation Experiment

A panel of eight DEG-transcript-probes, namely *FABP3* (Fatty acid-binding protein 3), *IL1B* (*Interleukin 1B*), *ITGAV* (*Integrin AV*), *NMB* (*Neuromedin B*), *OPN* (*Osteopontin*), *PPARG* (*Peroxisome proliferator-activated gamma receptor*), *RBP4* (Retinol Binding Protein) and *S100A11* (S100A11 protein) were selected to validate the microarray experimental results (Table 3). Selected DEG-transcript-probes were associated with regulation of significant biologic processes, such as regulation of immune response (*IL1B* and *S100A11*), angiogenesis (*IL1B*), lipid metabolism (*FABP3 and PPARG*), cell adhesion (*ITGAV* and *OPN*), intercellular transmission (*NMB*) and response to stimuli (*RBP4*).

#### 2.5.5. Statistics of Expression of Selected Genes in the Endometrium of Pregnant Gilts

Raw data on gene expression were converted in the LC480 conversion program (HFRC, the Netherlands), and then using the LinRegPCR 12.10 application (HFRC, the Netherlands) [38] the reaction efficiency was determined individually for each sample and each amplicon. Relative expression levels were determined based on differences in Cp points (ΔCp) with correction of amplification efficiency (E). Using the following mathematical model:

Ratio = (E_target_)^−ΔCp target (control-sample)^/(E_ref_)^−ΔCp ref (control-sample)^

Expression analysis results were normalized based on two reference genes (geometric mean of two genes). The significance of differences in the relative level of gene expression between the analyzed groups was determined based on the Kruskal–Wallis test.

## 3. Results

### 3.1. Principle Component Analysis (PCA)

**Figure 3:**
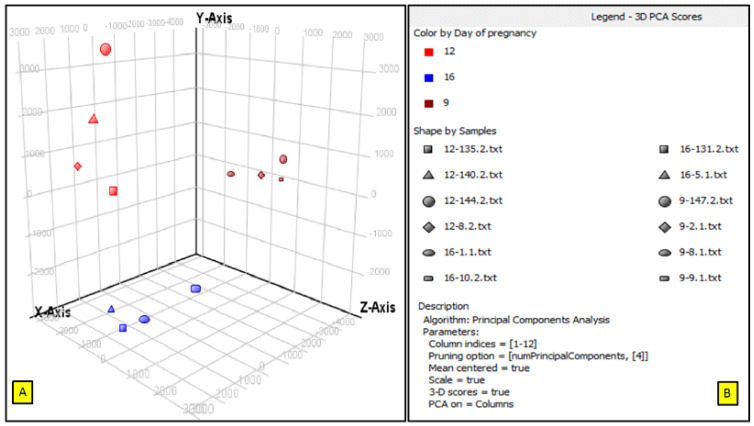
Principal component analysis of the early stages of pregnancy in PLW gilts. (**A**) The principal component analysis for three stages of early pregnancy in microarray data. Each symbol in X, Y and Z axis indicates a sample. (**B**) A legend explains principal component analysis (PCA) scores of three stages of early pregnancy in PLW gilts. The red, blue and dark red squares represent the endometrial samples of 9D, 12D and 16D of early pregnancies.

The quality analysis of endometrial microarray data were represented by the grouping of early stages of pregnancies based on the expression signals across analyzed endometrial transcriptome samples. The PCA results revealed the characteristics of the grouping of all three stages of early pregnancies in PLW gilts based on their expression signals across analyzed endometrial transcriptome samples (Figure 3, Table S1).

### 3.2. Comparisons of Porcine Endometrium at Three Early Pregnancy Periods in PLW Gilts

The DEG-transcript-probes were identified by comparing three stages (9D, 12D and 16D, respectively) of early pregnancy in PLW gilts. The DEG-transcript-probes were identified without any cutoff values. Microarray gene expression profile representing all transcript probes (Tables S2–S4) were filtered according to two stringent criteria using Microsoft Excel; (i) the significant DEG-transcript-probes with cutoff *p* values of *p* < 0.001 and >2 FC; and (ii) the significant blastn E-values and high-scoring segment pair (HSP) values of microarray probes sequences against representatives of gene-transcript of *sus scrofa* assembly *Sscrofa11.1* [26].

#### 3.2.1. Comparisons of Porcine Endometrium 9D vs. 12D of the Early Pregnancy Period

Comparison of the porcine endometrium 9D vs. 12D of early pregnancy period (cutoff values of *p* < 0.001 and >2 FC) revealed a total of 6049 significant DEG-transcript-probes, including 3665 upregulated and 2384 downregulated (Table S5). Furthermore, after nucleotide-nucleotide BLAST (blastn) of microarray probes sequences against representatives of gene-transcript of Sus scrofa, a total of 4097 significant DEG-transcript-probes (cutoff E-values of < 0.06 and >2 FC) were identified. Selected set includes 2357 upregulated and 1740 downregulated (Table S6).

#### 3.2.2. Comparison of Porcine Endometrium 9D vs. 16D of the Early Pregnancy Period

Comparison of porcine endometrium 9D vs. 16D of early pregnancy period (cutoff values of *p* < 0.001 and >2 FC), revealed a total of 374 significant DEG-transcript-probes, including 184 upregulated and 190 downregulated (Table S7). In addition, blastn of microarray probes sequences against representatives of gene-transcript of *Sus scrofa* assembly *Sus scrofa11.1*, allowed the identification of 197 significant DEG-transcript-probes (cutoff E-values of < 0.06 and >2 FC), including 103 upregulated and 94 downregulated (Table S8).

#### 3.2.3. Comparison of Porcine Endometrium 12D vs. 16D of the Early Pregnancy Period

Results for 12D vs. 16D of early pregnancy period (cutoff values of *p* < 0.001 and >2 FC) were also compared. As a result, total of 6034 significant DEG-transcript-probes were identified, including 2166 upregulated and 3868 downregulated (Table S9). Furthermore, after blastn of microarray probes sequences against representatives of gene-transcript of *Sus scrofa* assembly *Sscrofa11.1*, a total of 4066 significant DEG-transcript-probes (cutoff E-values of < 0.06 and >2 FC) were identified, including 1567 upregulated and 2499 downregulated (Table S10).

### 3.3. Comparison of Upregulated and Downregulated DEG-transcript-probes at Three Stages of Early Pregnancy in PLW Gilts

The comparisons and interpretations of upregulated and downregulated DEG-transcript-probes at three stages of early pregnancy in PLW gilts were performed using Venn diagrams [39].

#### 3.3.1. Comparison of Upregulated DEG-transcript-probes during Three Stages of Early Pregnancy

**Figure 4:**
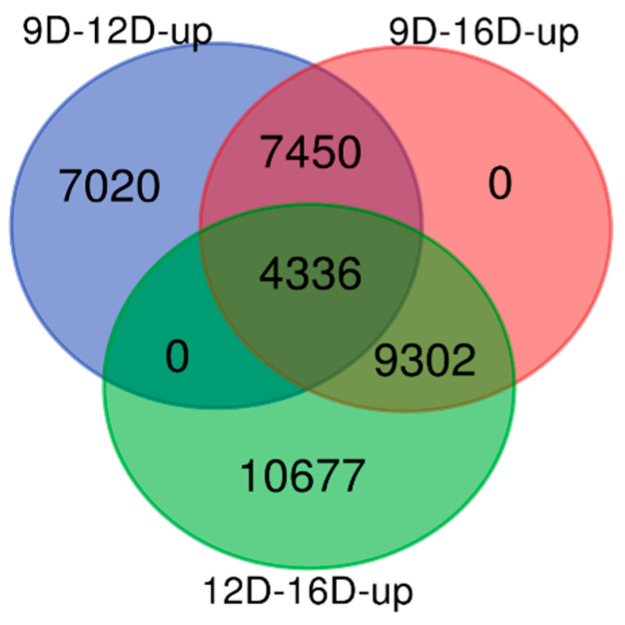
Identification of pregnancy-stage specific upregulated differentially expressed gene (DEG)-transcript-probes without cutoff *p*-values by comparing endometrium transcriptome at 9th day (9D) versus 12th day (12D) versus 16th day (16D) of early pregnancy in PLW gilts. The numeric values (n) of the Venn diagram representing the endometrial DEG-transcript-probes listed in Tables S2–S4.

Compilation of results for the upregulated DEG-transcript-probes (without cutoff *p*-values) (Figure 4), allowed for identification of 7020 and 10,677 endometrial DEG-transcript-probes that were termed as uniquely observed early pregnancy stage-specific DEG-transcript-probes in 9D vs. 12D and 12D vs. 16D, respectively. In contrast, not a single unique DEG-transcript was identified in 9D vs. 16D DE-comparison. Moreover, a total of 7450 and 9302 upregulated endometrial DEG-transcript-probes were commonly shared in 9D vs. 12D and 12D vs. 16D. In contrast, not a single DE gene-transcript was shared in 9D vs. 16D DE-comparison. Finally, a total of 4336 upregulated endometrial DEG-transcript-probes were commonly shared among all three comparisons of endometrium transcriptome at early pregnancy periods (Figure 4).

**Figure 5:**
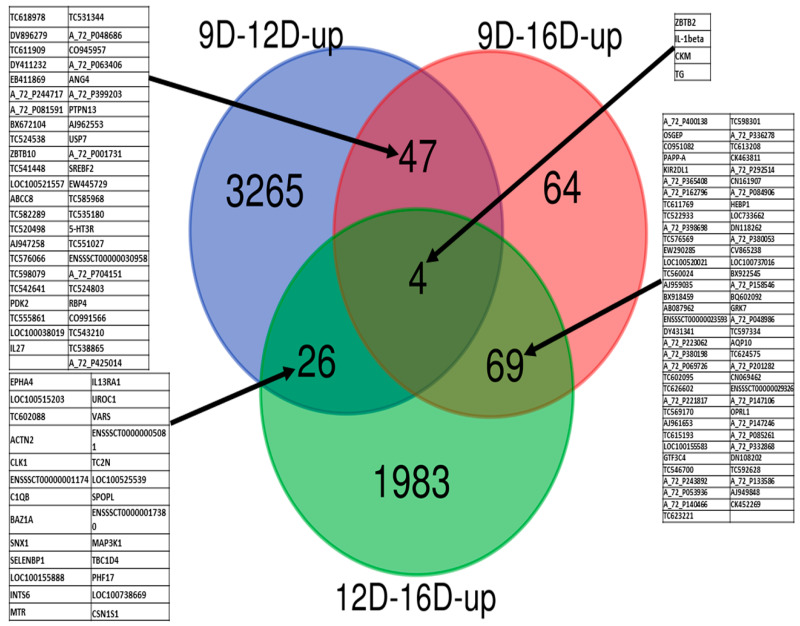
Identification of pregnancy-stage specific upregulated DEG-transcript-probes with cutoff *p*-values < 0.001 and >2 FC by comparing endometrium transcriptome at 9th day (9D) versus 12th day (12D) versus 16th day (16D) of early pregnancy in PLW gilts. The numeric values (n) of the Venn diagram representing the endometrial DEG-transcript-probes listed in Tables S5, S7 and S9. Transcript symbols of commonly sheared microarray-probes are indicated in the blocked tables.

Furthermore, comparison of the significant upregulated DEG-transcript-probes with cutoff values of *p* < 0.001 and >2 FC, at three stages of early pregnancy in PLW gilts (Figure 5), identified a total of 3265, 64 and 1983 endometrial DEG-transcript-probes as uniquely observed in 9D vs. 12D, 9D vs. 16D and 12D vs. 16D, respectively. A total of 47, 69 and 26 upregulated endometrial DEG-transcript-probes were commonly shared between 9D vs. 12D, 9D vs. 16D and 12D vs. 16D DE-comparisons, respectively. Highly significant upregulated endometrial DEG-transcript-probes were also identified as commonly shared among all three comparisons of endometrium transcriptome during early pregnancy (Figure 5). These transcripts are involved in regulation of the anatomic structure development and transport (*thyroglobulin: TG*), *methyltransferase* activity, DNA binding, biosynthetic and cellular nitrogen compound metabolic process (*zinc finger and BTB domain containing-2: ZBTB2*), ion binding, *kinase* activity (*creatine kinase, M-type: CKM*), cell proliferation and differentiation, as well as *apoptosis* activity (*interleukin-1beta: IL1B*).

**Figure 6:**
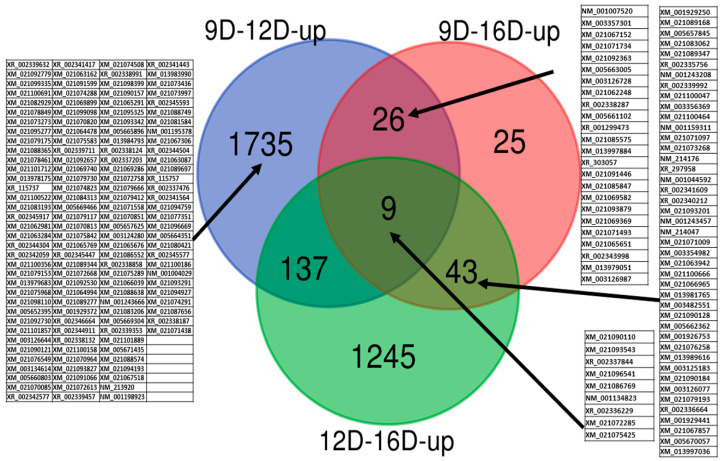
Identification of pregnancy-stage specific upregulated DEG-transcript-probes with cutoff E-values < 0.06 and >2 FC by comparing endometrium transcriptome at 9th day (9D) versus 12th day (12D) versus 16th day (16D) of early pregnancy in PLW gilts. The numeric values (n) of the Venn diagram representing the endometrial DEG-transcript-probes listed in Tables S6, S8 and S10. Blasted transcript accession numbers of commonly sheared microarray-probes are indicated in blocked tables.

The juxtaposition of the blastn of microarray probes sequences against representatives of gene-transcript (*Sus-scrofa* assembly *Sscrofa11.1* using CLC, genomics) at three stages of early pregnancy in PLW gilts (Figure 6), identified as uniquely observed a total of 1735, 25 and 1245 upregulated endometria DEG-transcript-probes (E-values < 0.06 and >2 FC) in 9D vs. 12D, 9D vs. 16D and 12D vs. 16D, respectively. Furthermore, a total of 26, 43 and 137 upregulated endometrial DEG-transcript-probes were commonly shared between 9D vs. 12D, 9D vs. 16D and 12D vs. 16D DE-comparisons, respectively. Nine of the highly significant upregulated endometrial DEG-transcript-probes identified as commonly shared among all three comparisons of endometrium transcriptome during early pregnancy (Figure 6) regulates the *methyltransferase* activity, DNA binding, biosynthetic and cellular nitrogen compound metabolic process (*ZBTB2*), ion binding, *kinase* activity (*CKM*), anatomic structure development, reproduction and growth (*selenoprotein P: SELENOP*), cytoskeletons organization, signal transduction, ion binding and kinase activity (*cell-division cycle-42 binding protein kinase alpha: CDC42BPA*), phosphatase activity(*multiple inositol-polyphosphate phosphatase 1: MINPP1*), enzyme binding, signal transduction response to stress, immune system process, cell population proliferation (*guanine nucleotide exchange factor-3: VAV3*), cancer susceptibility candidate gene (*cancer susceptibility candidate gene 4 protein: CASC4*), cytoskeletal protein binding (*cordon-bleu WH2 repeat protein-like 1: COBLL1*) and enzyme regulator activity, ion binding, (*ArfGAP with coiled-coil, ankyrin repeat and PH domains-2: ACAP2*).

#### 3.3.2. Comparison of Downregulated DEG-transcript-probes during Three Stages of Early Pregnancy

**Figure 7:**
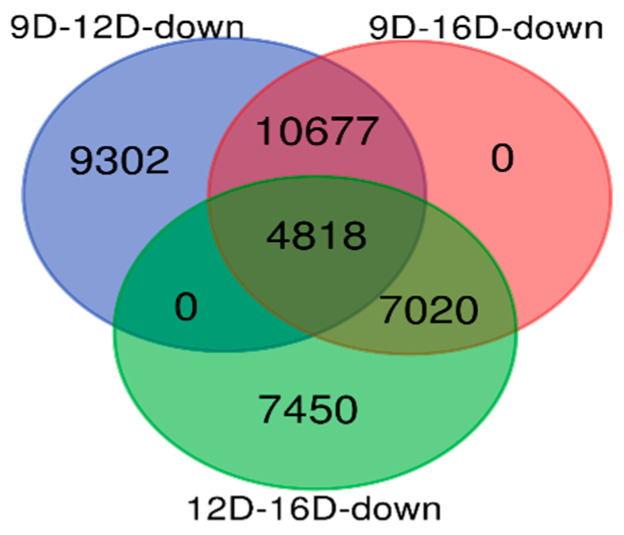
Identification of pregnancy-stage specific downregulated DEG-transcript-probes without cutoff *p*-values by comparing endometrium transcriptome at 9th day (9D) versus 12th day (12D) versus 16th day (16D) of early pregnancy in PLW gilts. The numeric values (n) of the Venn diagram representing the endometrial DEG-transcript-probes are listed in Tables S2–S4.

Comparison of the downregulated DEG-transcript-probes without cutoff *p*-values (Figure 7), revealed a total of 9302 and 7450 endometrial DEG-transcript-probes that were identified as uniquely observed in 9D vs. 12D and 12D vs. 16D. In contrast, not a single unique DEG transcript was identified in 9D vs. 16D. Moreover, a total of 10,677 and 7020 downregulated endometrial DEG-transcript-probes were commonly shared at 9D vs. 12D and 12D vs. 16D of DE comparisons. In contrast, not a single DE gene transcript was shared at 9D vs. 16D DE-comparison. Lastly, a total of 4818 downregulated endometrial DEG-transcript-probes were commonly shared among all three comparisons of endometrium transcriptome during early pregnancy (Figure 7).

**Figure 8:**
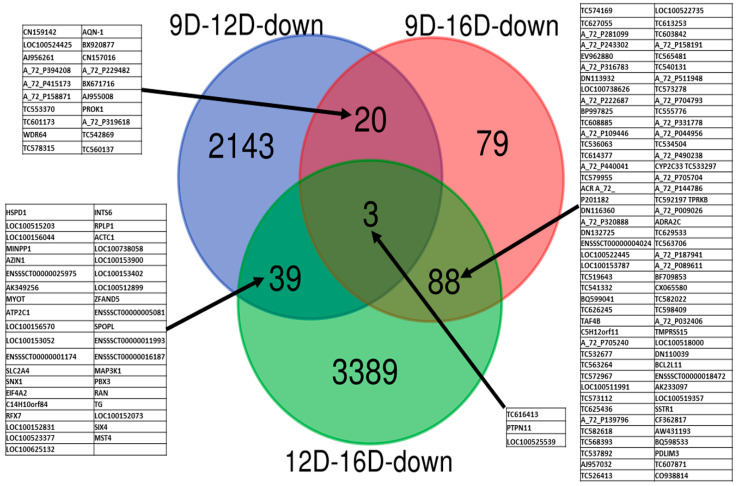
Identification of pregnancy-stage specific downregulated DEG-transcript-probes with cutoff *p*-values < 0.001 and >2 FC by comparing endometrium transcriptome at 9th day (9D) versus 12th day (12D) versus 16th day (16D) of early pregnancy in PLW gilts. The numeric values (n) of the Venn diagram representing the endometrial DEG-transcript-probes are listed in Tables S5, S7 and S9. Transcript symbols of commonly sheared microarray-probes are indicated in the blocked tables.

Analysis of data for the significant downregulated DEG-transcript-probes at three stages of early pregnancy in PLW gilts (Figure 8), obtained after application of cutoff values of *p* < 0.001 and >2 FC, revealed a total of 2143, 79 and 3389 endometrial DEG-transcript-probes, identified as uniquely observed in 9D vs. 12D, 9D vs. 16D and 12D vs. 16D, respectively. Moreover, a total of 20, 88 and 39 downregulated endometrial DEG-transcript-probes were commonly shared at 9D vs. 12D, 9D vs. 16D and 12D vs. 16D DE-comparisons, respectively. Three of the downregulated endometrial DEG-transcript-probes identified as commonly shared among all three comparisons of endometrium transcriptome during early pregnancy (Figure 8) are known to regulate the cellular protein modification process and phosphatase activity (*protein tyrosine phosphatase non-receptor type 11: PTPN11*).

**Figure 9:**
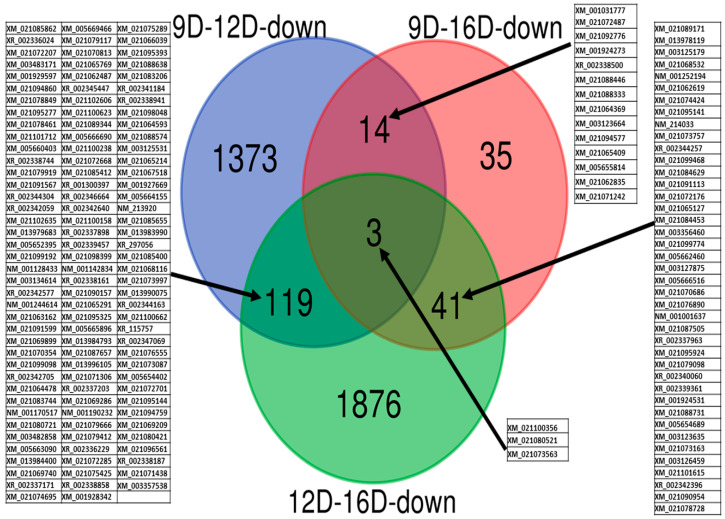
Identification of pregnancy-stage specific downregulated DEG-transcript-probes with cutoff E-values < 0.06 and >2 FC by comparing endometrium transcriptome at 9th day (9D) versus 12th day (12D) versus 16th day (16D) of early pregnancy in PLW gilts. The numeric values (n) of the Venn diagram representing the endometrial DEG-transcript-probes are listed in Tables S6, S8 and S10. Blasted transcript accession numbers of commonly sheared microarray-probes are indicated in blocked tables.

The approach of using the blastn tool for comparison of microarray probes sequences against representatives of gene-transcript of *Sus scrofa* assembly *Sscrofa11.1* (using CLC, genomics) at three stages of early pregnancy in PLW gilts was also used for downregulated DEG-transcript-probes (Figure 9). A total of 1373, 35 and 1876 downregulated endometrial DEG-transcript-probes (E < 0.06 and >2 FC) was identified as uniquely observed at 9D vs. 12D, 9D vs. 16D and 12D vs. 16D, respectively. Furthermore, a total of 14, 41 and 119 downregulated endometrial DEG-transcript-probes were commonly shared between 9D vs. 12D, 9D vs. 16D and 12D vs. 16D DE-comparisons, respectively. Three of highly significant downregulated endometrial DEG-transcript-probes identified as commonly shared among all three comparisons of endometrium transcriptome during early pregnancy (Figure 9) regulates the cellular protein modification process and *phosphatase* activity (*PTPN11*), cellular protein modification process, catabolic process, response to stress, cellular nitrogen compound metabolic process, DNA metabolic process (*Kelch-like family member 15: KLHL15*), response to stress and signal transduction (*transmembrane protein 33: TMEM33*).

### 3.4. Comparison and the Heatmap Visualization of the Porcine Endometrium at 12D vs. 16D vs. 16D of early Pregnancy Period

**Figure 10:**
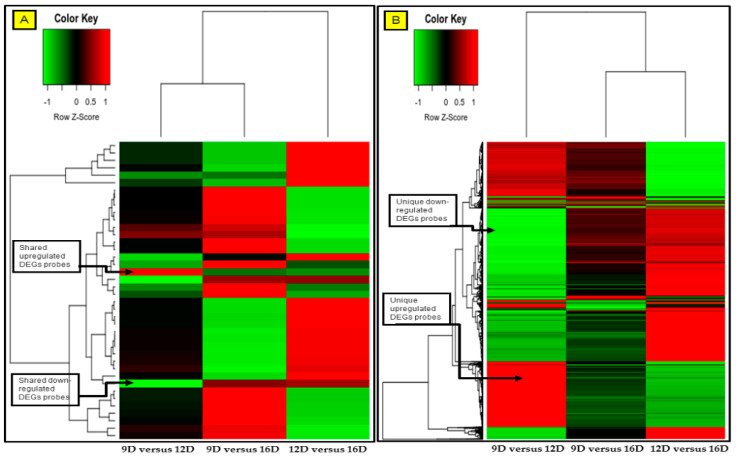
Heatmap comparisons at 9D versus 12D versus 16D of early pregnancy in PLW gilts. Figure showing the gene expression profile of (A) common shared and (B) unique pregnancy-stage specific upregulated (red) and downregulated (green) DEG-transcript-probes with cutoff *p*-values < 0.001 and >2 FC. The DEG-transcript-probes with similar expression profiles were clustered together by hierarchical clustering. The numeric values (n) of the heatmap representing the endometrial DEG-transcript-probes probes are listed in Tables S5, S7 and S9.

**Figure 11:**
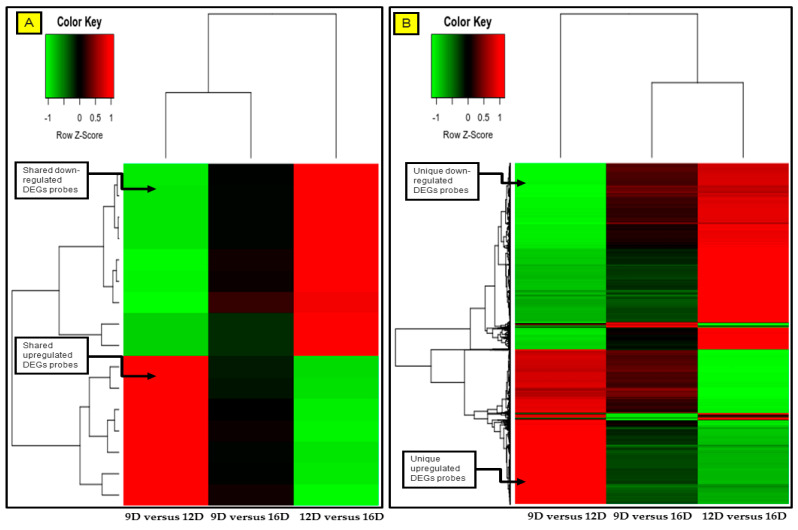
Heatmap comparisons at 9D versus 12D versus 16D of early pregnancy in PLW gilts. Figure showing the gene expression profile of (**A**) common shared and (**B**) unique pregnancy-stage specific upregulated (red) and downregulated (green) DEG-transcript-probes with cutoff E-values < 0.06 and >2 FC. The DEG-transcript-probes with similar expression profiles were clustered together by hierarchical clustering. The numeric values (n) of the heatmap representing the endometrial DEG-transcript-probes probes are listed in Tables S6, S8 and S10.

The gene expression profiles of upregulated and downregulated endometrial DEG-transcript probes were visualized using the heatmap. Based on the Tables S5, S7 and S9, the heatmap was generated for the significant DEG-transcript-probes with cutoff *p* values of *p* < 0.001 and >2 FC (Figure 10). Furthermore, the heatmap was generated with blastn E-values and >2 FC based on the Tables S6, S8 and S10, for the significant DEG-transcript-probes (Figure 11).

### 3.5. Comparison and Visualization of Gene Network and Pathways using ClueGO of Porcine Endometrium at 12D vs. 16D vs. 16D of Early Pregnancy Period

#### 3.5.1. Visualization of Gene Network and Pathways of Porcine Endometrium at 9D vs. 12D of Early Pregnancy Period

**Figure 12:**
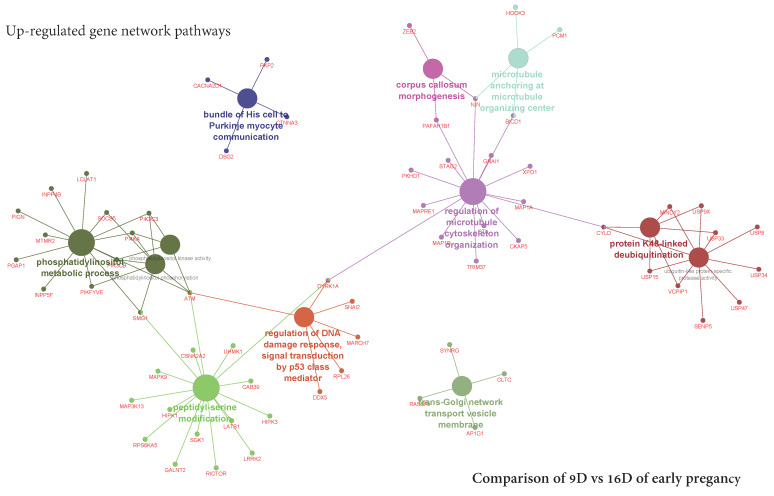
ClueGO upregulated network of pathways by comparing 9D vs. 12D of early pregnancy. Each node represents a GO biologic process and the colors represent the GO group (Table S12). The enrichment significance of pathway is reflected by the size of the nodes.

**Figure 13:**
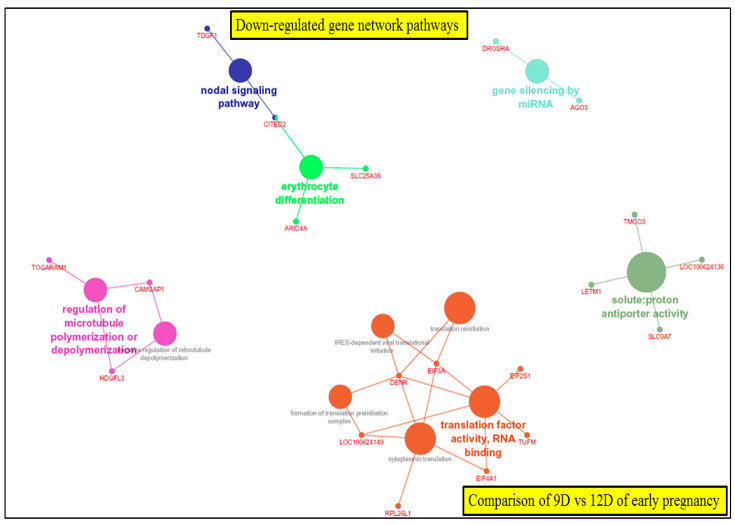
ClueGO downregulated network of pathways by comparing 9D vs. 12D of early pregnancy. Each node represents a gene ontology (GO) biologic process and the colors represent the GO group. The enrichment significance of pathway is reflected by the size of the nodes.

**Figure 14:**
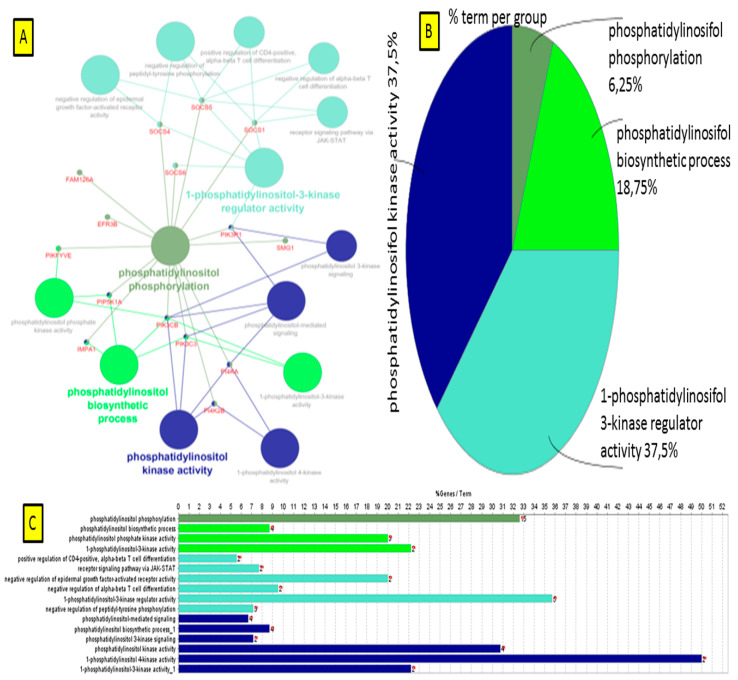
ClueGO analysis of the upregulated gene-network responsible for phosphatidylinositol metabolic process. (**A**) The most important biologic processes enriched by phosphatidylinositol pathway is given as a network in different colors; (**B**) molecular function and cellular components of phosphatidylinositol pathway are given in the form of a pie-chart; (**C**) various KEGG metabolic pathways enriched by phosphatidylinositol metabolic process.

The KEGG pathway analysis (ClueGO network of pathways) indicated that the predicted target upregulated genes (Tables S12 and S13) were mainly involved in gene-networks responsible for phosphatidylinositol metabolic process, peptidyl serine modification, regulation of DNA damage, corpus callosum *morphogenesis*, regulation of microtubule cytoskeletons organization, protein K48 linked deubiquitination and trans-Golgi network transport vesicle membrane (Figure 12, Table S12). In turn, downregulated genes took part in gene-networks responsible for nodal signaling, erythrocyte differentiation, regulation of microtubules polymerization, translation factor activity and solute proton antiporter activity pathway (Figure 13, Table S13). Additionally, the gene network and pathway of upregulated DEGs-transcripts probes showing the higher activity of genes in phosphatidylinositol phosphorylation (EFR3B, FAM126A, IMPA1, PI4K2B, PI4KA, PIK3C3, PIK3CB, PIK3R1, PIKFYVE, PIP5K1A, SMG1, SOCS1, SOCS4, SOCS5, SOCS6) was individually performed on ClueGO and presented in Figure 14.

#### 3.5.2. Visualization of Gene Network and Pathways of Porcine Endometrium at 9D vs. 16D of Early Pregnancy

**Figure 15:**
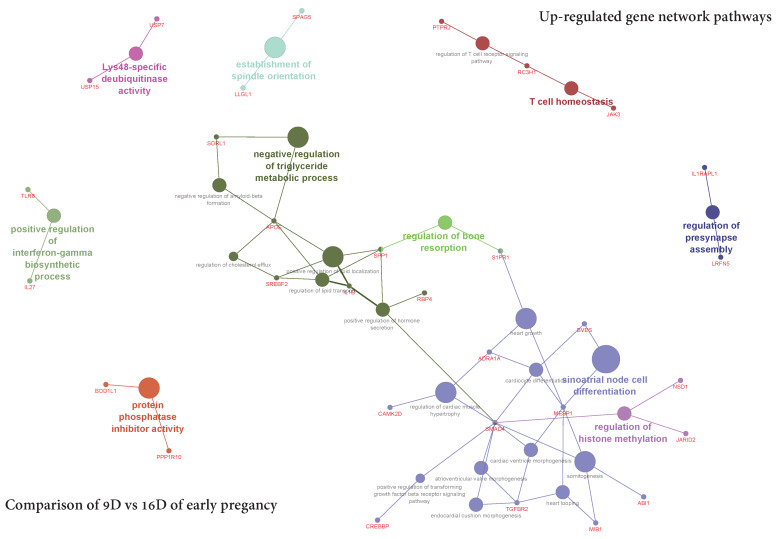
ClueGO upregulated genes network of pathways by comparing 9D vs. 16D of early pregnancy. Each node represents a GO biologic process and the colors represent the GO group. The enrichment significance of pathway is reflected by the size of the nodes.

**Figure 16:**
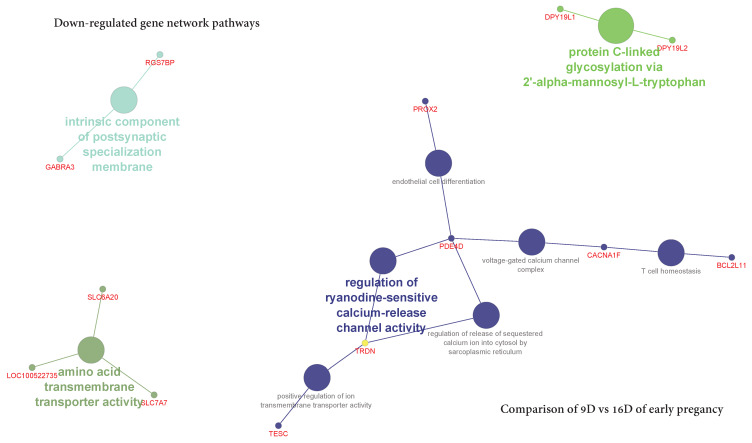
ClueGO downregulated genes network of pathways by comparing 9D vs. 16D of early pregnancy. Each node represents GO biologic process and the colors represent the GO group. The enrichment significance of pathway is reflected by the size of the nodes.

The ClueGO network of pathways analysis indicated that the predicted target upregulated genes (Table S14) were mainly involved in gene-networks responsible for Lys48-specific deubiquitinase activity, the establishment of spindle orientation, positive regulation of interferon-gamma biosynthetic process, positive regulation of transforming growth factor beta receptor signaling pathway, negative regulation of triglyceride metabolic process, protein phosphatase inhibitor activity, regulation of bone resorption, regulation of presynapse assembly, T cell homeostasis, sinoatrial node cell differentiation and regulation of histone methylation” (Figure 15, Table S14). Similarly, the predicted target downregulated genes (Table S15) took part in gene-networks responsible for amino acid transmembrane transporter activity, protein C-linked glycosylation via 2’-alpha-mannosyl-L-tryptophan, intrinsic component of postsynaptic specialization membrane, T cell homeostasis, endothelial cell differentiation, voltage-gated calcium channel complex, positive regulation of ion transmembrane transporter activity, regulation of release of sequestered calcium ion into the cytosol by the sarcoplasmic reticulum and regulation of ryanodine-sensitive calcium-release channel activity (Figure 16**,** Table S15).

#### 3.5.3. Visualization of Gene Network and Pathways of Porcine Endometrium at 12D vs. 16D of Early Pregnancy Period

**Figure 17:**
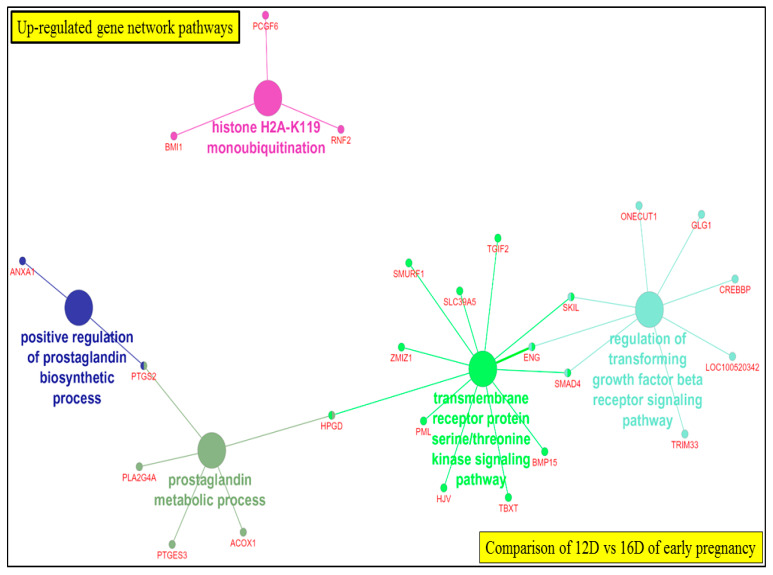
ClueGO upregulated genes network of pathways by comparing 12D vs. 16D of early pregnancy. Each node represents a GO biologic process and the colors represent the GO group. The enrichment significance of pathway is reflected by the size of the nodes.

**Figure 18:**
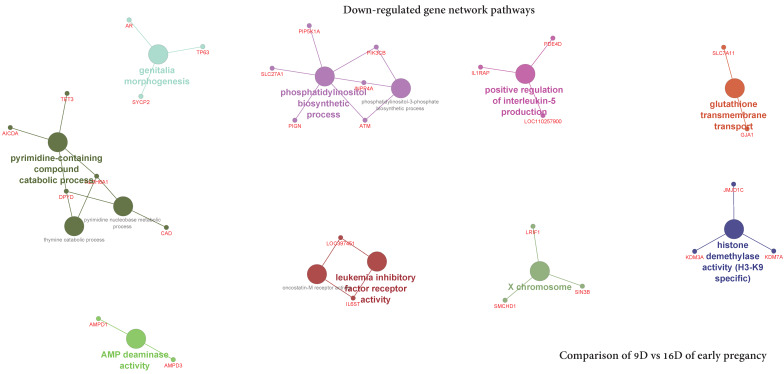
ClueGO downregulated genes network of pathways by comparing 12D vs. 16D of early pregnancy. Each node represents a GO biologic process and the colors represent the GO group. The enrichment significance of pathway is reflected by the size of the nodes.

The ClueGO network of pathways analysis indicated that the predicted target upregulated genes (Table S16) were involved in gene-networks responsible for prostaglandin metabolic process, transmembrane receptor protein serine/threonine kinase signaling pathway, regulation of transforming growth factor beta receptor signaling pathway, positive regulation of prostaglandin biosynthetic process and histone H2A-K119 monoubiquitination (Figure 17, Table S16). Similarly, the predicted target downregulated genes (Table S17) took part in gene-networks responsible for X chromosome, AMP deaminase activity, genitalia morphogenesis, histone demethylase activity (H3-K9 specific), positive regulation of interleukin-5 production, glutathione transmembrane transport, phosphatidylinositol biosynthetic process, phosphatidylinositol-3-phosphate biosynthetic process, oncostatin-M receptor activity, leukemia inhibitory factor receptor activity, pyrimidine-containing compound catabolic process, pyrimidine nucleobase metabolic process and thymine catabolic process (Figure 18**,** Table S17).

### 3.6. Validation of Microarray Experiment using RT-PCR

**Figure 19:**
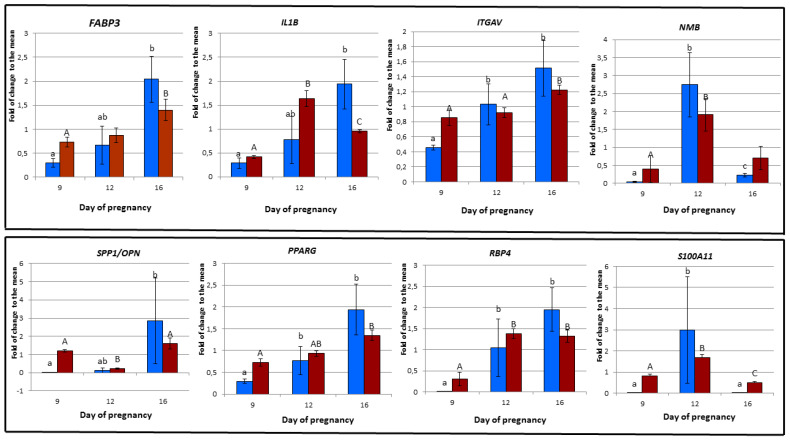
Validation of pregnancy-specific porcine endometrial microarray experiment via RT PCR. Figure axis X denotes the endometrium transcriptome at 9th day (9D), 12th day (12D) and 16th day (16D) of early pregnancy in PLW gilts. Figure Y-axis denotes the gene expression change as fold of change (FC) expression relative to mean value of particular DEG-transcript in porcine endometrial tissue determined via microarray technic (Dark red bar) and RT PCR (Blue bar). The same letter indicates a significant difference: lowercase *p* < 0.05, uppercase *p* < 0.01. The different letter indicates a significant difference: *p* < 0.05; lowercase RT-PCR, uppercase microarrays. “ab” or “AB” indicate no significant difference from “a” and “b” or “A” and “B”, respectively.

Obtained results of the RT-PCR analysis allowed to confirm the microarray expression profiles both in terms of its size and direction of change. In both methods, microarray and RT-PCR, particular gene expression was compared against the overall mean value of 9D, 12D and 16D of early pregnancy and presented as fold of change (FC) relative to mean (Table 4 and Table 5). The most considerable differences were observed in the gene expression level of *ITGAV* and *S100A11* genes at 12D and of *ITGAV* gene at 16D of early pregnancy. However, it should be pointed out that not a single gene with significant differences in the gene expression level was observed during the 9D of early pregnancy (Figure 19, Table S18). Almost all of the results of RT-PCR are in agreement to microarray experiment. Different trends of gene expression were found in case of *ITGAV* and *RBP4* genes, expressed at the 16D of early pregnancy.

## 4. Discussion

### 4.1. Identification of DEG Transcripts in Porcine Endometrium during Early Stages of Pregnancy

Early pregnancy is associated with dynamic changes in the porcine endometrium reflected by the changes of many genes’ expression. In pigs, the maternal recognition of pregnancy and embryo implantation occurs approximately at 12D of pregnancy and the presence of the conceptus in the uterine lumen during this period changes the uterine endometrial function to prepare for attachment of the conceptus to the endometrial epithelial cells and maintain luteal function in the ovary [40]. In this study, a broad set of putative significant upregulated and downregulated DEG-transcript-probes (*p* < 0.001 and >2 FC), in all three comparisons, were identified (Tables S2–S10).

In the past, several studies based on global comparisons of the porcine endometrial transcriptome during the different stages of early pregnancy were reported to identify the DEGs in the uterine endometrium at 12D and 14D of pregnancy [41,42]. However, majority of such studies investigated the endometrial DEG differences between pregnancy (pregnant) and estrus cycle (non-pregnant) to show the global pattern of endometrial gene expression that varied during early pregnancy [13,43,44,45]. Analyzing 23,937 microarray gene-probes, Kim et al. 2012 [13], identified 99 and 213 upregulated DEGs, 92 and 231 downregulated DEGs in the uterine endometrium by comparison of D12 and D15 of pregnancy vs. D12 and D15 of the estrous cycle. The functional annotation study revealed that DEGs are involved in the regulation of immunity, steroidogenesis, cell-to-cell interaction and tissue remodeling activities. In another study by Kim et al. 2015 [43], a total of 6,991 DEGs were identified by comparing genes expressed at 12D of pregnancy with those at D15, D30, D60, D90 and D114 of pregnancy. Several pregnancy-related hub genes such as *ALPPL2, RANBP17, NF1B, SPP1 and CST6* were discovered through network analysis. In a study based on Affymetrix GeneChip porcine genome array, Østrup et al. 2010 [44] identified 263 genes to be significantly differentially expressed between the pregnant and non-pregnant sows during the time of initial placentation, i.e., at 14D after insemination. However, comparison of the pregnancy vs. estrous cycle transcriptomic profile of pig endometrium at 12D and 16D of the gestation, by Kiewisz et al. [45], revealed 110 (12D) and 179 (16D) DEGs, respectively. Microarray data analysis showed a total of 266 upregulated and 323 downregulated DEGs in the endometrium harvested during early pregnancy compared to the endometrium during the estrous cycle. Moreover, comparison of pregnancy vs. estrous cycle transcriptomic profile of pig endometrium further identified the estrogen, transforming growth factor (TGF) β1 and fibroblast growth factor (FGF2) regulating genes at 12D of gestation and the epidermal growth factor (EGF), insulin and interleukin 11 (IL-11) regulating genes at 16D of gestation [45].

In the past several breed-specific DEGs in porcine endometrium during early pregnancy were also reported [6,46]. For instance, the comparison of endometrium at 12D of early pregnancy between Meishan and Yorkshire [6] allowed to find 17,076 microarray probe-sets in at least two Affymetrix arrays and a total of 14,951 and 14,558 DEG-transcript-probes were identified in Meishan and Yorkshire endometrium respectively. In our study, we have found 43,603 probe-sets “present” in at least all three pooled arrays and a total of 6049 (3665 upregulated and 2384 downregulated) (Table S5), 374 (184 upregulated and 190 downregulated) (Table S7) and 6034 (2166 upregulated and 3868 downregulated) (Table S9), during 9D vs. 12D, 9D vs. 16D and 12D vs. 16D of early pregnancy in PLW gilts. In another study on breed-specific gene expression comparison of the endometrium [46], during early gestation [15D] vs. mid-gestation period [26D and 50D], revealed a total of 689 and 1649 annotated genes to be differentially expressed in Meishan and Yorkshire endometrium. This study allowed further annotation of DEGs and 73 of them were unique to Meishan endometrium, 536 were unique to Yorkshire endometrium and 228 DEGs were common in Meishan and Yorkshire endometrium. In this study, unique upregulated and downregulated DEG-transcript-probes in all three comparisons of PLW gilts were also identified. A total of 3265, 64 and 1983 upregulated, as well as 2143, 79 and 3389 downregulated endometrial DEG-transcript-probes were uniquely observed at 9D vs. 12D, 9D vs. 16D and 12D vs. 16D, of early pregnancy in PLW gilts (Figure 1, Figure 2, Figure 3, Figure 4, Figure 5 and Figure 6). After the GO term enrichment analysis, upregulated DEG-transcript-probes during 9D, 12D and 16D of early pregnancy were found to be responsible for the anatomic structure development and transport (*TG*), DNA binding, biosynthetic and cellular nitrogen compound metabolic process and *methyltransferase* activity (*ZBTB2*), ion binding and *kinase* activity (*CKM*), cell proliferation, differentiation and *apoptosis* activity (*IL1B*), anatomic structure development, reproduction and growth (*SELENOP*), cytoskeleton organization and signal transduction, (*CDC42BPA*), cytoskeletal protein binding (*COBLL1*), *phosphatase* activity (*MINPP1*), enzyme binding, signal transduction response to stress, immune system process, cell population proliferation (*VAV3*), cancer susceptibility candidate gene (*CASC4*) and enzyme regulator activity and ion binding (*ACAP2*). The binding of ions, enzymes, DNA and other binding proteins regulated by *CKM, ACAP2, CDC42BPA* and *ZBTB2* play a significant role in embryo implantation during early pregnancy [47,48,49]. Previous studies suggested that the proper action of ion channels is not only essential to regulate the volume of fluid in the uterine lumen, but also for the regulation of the genes responsible for implantation of the embryo [50]. In contrast, abnormalities in binding and transport of ions in the uterus may be associated with disorders of receptivity and implantation of the embryo [51]. In a recent RNA-seq study [52], based on transcriptome analysis of the endometrium from Chinese Erhualian sows at 12D of early pregnancy, the concentration of calcium ion binding and cell adhesion in the highly prolific Erhualian sows (EH) compared to those of less prolific (EL) sows were significantly higher. In another recent study of Jalali et al. [53] expression and distribution of actin-binding proteins (ABPs) in porcine endometrium at 10D and 13D (preimplantation period) and 16D (attachment phase) of the estrous cycle or pregnancy had no effects on the ABP. However, the protein abundance of vinculin was significantly higher at 13D than 10D (*p* < 0.05) of the estrous cycle and its abundance was highest at 16D in the pregnant endometrium.

Results of the present study revealed over-expression of TG hormone and SELENOP genes associated with the anatomic structure development at the state of uterine receptivity and growth during early pregnancy. Similarly, the previous studies also suggested, that the expression of *type 2 iodothyronine deiodinase* in the rat uterus was induced during pregnancy [54]. In contrast, the gene expression of SELENOP was observed in uterus and placenta during late pregnancy [55] playing a significant role in female reproductive function during pregnancy [56].

Based on the present study, it could be noted that over-expression of *VAV3* and *CASC4* genes in PLW gilts during early pregnancy was related to the regulation of Rho/Rac family *GTPases* and cancer susceptibility activities. Both can have a significant role in determining endometrial cancer and served as a prognostic and predictive marker for endometrial cancer [57] and breast cancer [58]. Moreover, the study of Samborski et al. [41] also indicated that similar down-regulation of genes related to the immune response at 14D of early pregnancy, suggesting and confirming the importance of regulation of immunological processes. The results of the presented study showed, that several genes regulating cellular protein modification process and *phosphatase* activity (*PTPN11)*, catabolic process, response to stress, cellular nitrogen compound metabolic process and DNA metabolic process (*KLHL15*), response to stress and signal transduction (*TMEM33*) had reduced expression during 9D, 12D and 16D of early pregnancy in PLW gilts.

### 4.2. Identification of Gene Networks and Pathways in Porcine Endometrium during Early Stages of Pregnancy

During early pregnancy, the porcine embryo undergoes first cell divisions in oviduct and then four cells stage blastocyst arrives in the uterus, followed by hatching from the zona pellucida at 7D [59]. The porcine conceptus changes rapidly from spherical to tubular, and then filamentous form by differentiation of trophoblast cells making it ready for implantation between 10D and 12D [60,61]. During 12D and 14D of pregnancy, the porcine embryos appose and subsequently attach to the uterine luminal epithelium with evidence of the pronounced vascularization of the endometrium [42]. By comparing the 9D vs. 12D vs. 16D of pregnancy in PLW gilts, there were identified several upregulated and downregulated gene networks and pathways discussed below.

### 4.3. Genes Related to Signaling and Metabolism

The present study identified upregulated genes involved in phosphatidylinositol metabolic process and peptidyl serine modification suggesting that there may be selective activation of the *kinase* signaling and metabolic processes in the local embryonic environment during the early stages of pregnancy. Moreover, upregulated genes involved in the regulation of DNA damage and microtubule cytoskeleton organization, cell communication and endometrial remodeling, corpus callosum morphogenesis, protein K48 linked deubiquitination and trans-Golgi network transport vesicle membrane further suggested distinct participation of gene expression regulation during the early stages of pregnancy. Endometrial secretions responsible for cell type-specific signaling and metabolism during early stages of pregnancy driven by ovarian progesterone and conceptus signals are essential for conceptus growth and development. A recent study of Zeng et al. [7] revealed, that conceptus signals induce different transcriptomic regulations in the endometrial compartments/cell types related to their specific function during recognition and establishment of pregnancy. Their study identified various distinct upregulated genes functions and pathways related to estrogen signaling pathway, embryo development and cell proliferation in the luminal epithelium of pregnant gilts, as well as genes involved in sterol biosynthetic and metabolic processes and extracellular matrix in the stroma [7]. In our study, we have identified downregulated genes involved in nodal signaling, erythrocyte differentiation, regulation of microtubule polymerization, translation factor activity and solute proton antiporter activity pathways. These findings could suggest their essential role in the establishment and maintenance of the early stages of pregnancy. However, a recent study confirmed that gene networks of solute families encoding solute carrier family 8 member A3 and solute carrier family 24 members 4 genes were upregulated in the endometrium of Erhualian sows affecting embryonic survival rate during the peri-implantation period [52]. In our study, comparison between 12D and 16D identified upregulated gene-networks and pathways related to prostaglandin metabolic process, transmembrane receptor protein serine/threonine kinase signaling process, regulation of transforming growth factor beta receptor signaling process and positive regulation of prostaglandin biosynthetic process. Study of Kiewisz et al. [45] revealed, that gene networks related to the cell morphology, cell-to-cell signaling and interaction, cellular movement, skeletal and muscular system function and development and post-translational modification are the most common processes influenced by endometrial DEGs on 16D of pregnancy [45]. In our study, the most important finding is the identification of highly significant phosphatidylinositol phosphorylation gene networks and pathways in the comparison of 9D vs. 12D (upregulated) and 12D vs. 16D (downregulated). Very recently, the role of activation of phosphatidylinositol *3 kinase* (*PI3 K*) for embryo implantation during early pregnancy in the mouse model was described and confirmed by Su et al. 2020 [62]. Moreover, adiponectin significantly stimulated proliferation and suppressed apoptosis of porcine uterine luminal epithelial cells and would enhance uterine receptivity for embryo implantation. Study of Smolinska et al. 2019 [63] confirmed, that adiponectin-stimulated proliferation is related to activation of the *PI3 K.*

### 4.4. Genes Involved in Growth Factors and Immune Response

In our study, gene network and pathway involved in growth factor such as positive regulation of transforming growth factor beta receptor signaling pathway was overexpressed during 9D and 16D of early pregnancy. These findings indicate its potential role in the conceptus-endometrial interaction (embryo-maternal dialog) in context to the regulation of blood vessel growth, maturation and regression. In the study of Waclawik et al. 2017 [9], growth factor and growth factor receptor genes were found as differentially expressed in porcine endometrium during the establishment and implantation (preimplantation) phase of pregnancy [9]. In the present study, several upregulated gene networks and pathways related to the immune processes such as T cell homeostasis, sinoatrial node cell differentiation and positive regulation of interferon-gamma biosynthetic process were identified during early stages of pregnancy. During the pre-attachment phase (i.e., recognition of pregnancy and preparation for conceptus attachment) of early pregnancy, the unusually high overrepresentation of genes associated with T cell differentiation was reported [64]. In another study, pig endometrial immune cells (endometrial total T, T cytotoxic and T helper cells) were found in peak numbers during the attachment phase of implantation [65].

### 4.5. Genes Involved in Epithelial Cell Differentiation and Development

In our study, identified downregulated genes related to endothelial epithelium cell differentiation, amino acid transmembrane transporter activity, protein C-linked glycosylation via 2’-alpha-mannosyl-L-tryptophan and an intrinsic component of postsynaptic specialization membrane was identified between 9D and 16D of early pregnancy. Similarly, to our findings, the bioinformatics analysis of the obtained DEG revealed downregulation of functional categories related to epithelial cell differentiation in pregnant endometrium, particularly on 12D of pregnancy [45]. Their study revealed that epithelial growth factor (EGF) and its receptor (EGFR) were DE in porcine endometrium, where the EGF was downregulated in luminal epithelium and stroma and EGFR downregulated in the luminal epithelium [45]. Moreover, a study on cyclic and ovariectomised gilts demonstrated downregulation of endometrial EGFR expression in ovariectomised estradiol-treated gilts [66] and low abundance of EGFR mRNA in endothelial epithelium between 9D and 12D of pregnancy [67].

### 4.6. Genes Involved in Ion and Nutrient Transport

In our study, several gene networks and pathways involved in calcium channel complex, positive regulation of ion transmembrane transporter activity, regulation of release of sequestered calcium ion into the cytosol by the sarcoplasmic reticulum and regulation of ryanodine-sensitive calcium-release channel activity was identified. In a study of Zheng et al. 2018 [7], the ion transmembrane transport gene network showed the higher enrichment of upregulated genes in the endometrial stroma in comparison to genes upregulated in the luminal epithelium of pregnant gilts [7]. However, in another study, the calcium transporter genes had lower expression in endometrium luminal epithelium and stroma in pregnant gilts, particularly solute carrier family 24 member 4 (SLC24A4) [48]. The mRNAs for the calcium ion channel protein TRPV6 and the intracellular calcium-regulatory molecule S100G were upregulated in all three cell types (luminal epithelial cells, glandular epithelial cells and chorionic membrane), but highest in the luminal epithelium of pregnant gilts which is invalidated from quantitative PCR [68]. In our study, eight DEG transcript probes were validated by RT-PCR. The validated genes responsible for the regulation of immune response (IL1B, S100A11) [69,70], angiogenesis (IL1B) [70], lipid metabolism (FABP3 [71] and PPARG [16]), cell-adhesion (ITGAV [7]) and OPN [72]), intercellular transmission (NMB [73]) and response to stimuli (RBP4 [42]) genes were also confirmed by these previous studies [7,16,24,69,70,71,72,73].

## 5. Conclusions

This is the first study to report the comparison between three stages (9D, 12D and 16D) of early pregnancy in PLW gilts using microarray-based transcriptome analysis of endometrium tissue and provide an overall view of the complexity of the porcine endometrial transcriptome. Our results demonstrate that the microarray approach can be beneficial for identifying the microarray probes representing DE gene-transcripts during early pregnancy in PLW gilts. These identified and validated endometrial DEG transcripts can serve as useful genetic markers for reproduction and fertility trait-associated studies, to improve the genomic resources available for pigs, especially PLW breed. The study also provides the transcriptome evidence that demonstrates differences in the gene-transcripts expression between three different stages of early pregnancy of PLW gilts. In context to gene networks and pathways analysis, it can be concluded that identified phosphatidylinositol phosphorylation processes may have a significant role in the recognition and establishment of early pregnancy in PLW gilts.

## Figures and Tables

**Table 1:** Determination of corpus luteum at different stages of early pregnancies in the investigated Polish large white (PLW) gilts.

Number of Animals/sample ID	Day of Pregnancy	Number of Corpus Luteum in Right Ovary	Number of Corpus Luteum in Left Ovary
2.1	9	5	5
9.1	9	6	6
8.1	9	7	6
147.2	9	9	4
135.2	12	4	9
140.2	12	11	5
8.2	12	9	9
144.2	12	7	10
131.2	16	8	11
5.1	16	4	12
10.2	16	7	7
1.1	16	7	8

**Table 2:** Primer sequences of reference genes chosen for testing and selecting the reference genes for real-time PCR.

Gene	Full-Name	Primer Sequence	NCBI Access-Code
***TBP***	*TATA-Box Binding Protein*	F: 5’ - GATGGACGTTCGGTTTAGG- 3’ R: 5’ - AGCAGCACAGTACGAGCAA- 3’	DQ178122
***GAPDH***	*Glyceraldehyde-3-Phosphate Dehydrogenase*	F:5’-ACTCACTCTTCTACCTTTGATGCT-3’ R: 5’ - TGTTGCTGTAGCCAAATTCA - 3’	NM_001206359
***ACTB***	*Actin Beta*	F: 5’ - GGACTTCGAGCAGGAGATGG - 3’ R: 5’ - GCACCGTGTTGGCGTAGAGG - 3’	XM_003357928
***SDHA***	*Succinate Dehydrogenase Complex Flavoprotein Subunit A*	F: 5’ - GAACCGAAGATGGCAAGA - 3’ R: 5’ - CAGGAGATCCAAGGCAAA - 3’	XM_005659031
***HPRT1***	*Hypoxanthine Phosphoribosyltransferase 1*	F: 5’ - CCGAGGATTTGGAAAAGGT- 3’ R: 5’ - CTATTTCTGTTCAGTGCTTTG- 3’	NM_001032376
***CYTB***	*mitochondrially encoded cytochrome b*	F: 5’ - CACATCCAAACAACGAAGCA- 3’ R: 5’ -GTTCTACGGGTTGTCCTCCA - 3’	AY830188.1
***B2 M***	*beta-2-microglobulin*	F: 5’ - AAACGGAAAGCCAAATTACC- 3’ R: 5’ -ATCCACAGCGTTAGGAGTGA - 3’	XM_005659651
***YWHAZ***	*tyrosine 3-monooxygenase/tryptophan 5-monooxygenase activation protein zeta*	F: 5’ - ATGCAACCAACACATCCTATC- 3’ R: 5’ - GCATTATTAGCGTGCTGTCTT - 3’	XM_005662949
***HMBS***	*hydroxymethylbilane synthase*	F: 5’ - CTGTTTACCCAAGGAGCTGGA- 3’ R: 5’ - TGAAGCCAGGAGGAAGCA- 3’	NM_001097412

**Table 3:** Primer sequences of DEG-transcript-probes selected for validation by real-time PCR.

Gene	Primer Sequence	NCBI Access-Code
***FABP3***	F: 5’ - GGCCAACATGACCAAGCCTA - 3’ R: 5’ - CTGCCATGGGTGAGTGTCAG - 3’	NM_001099931
***RBP4***	F: 5’ - CCCCGAGGGACTCTTTCTGC - 3’ R: 5’ - TCTTGAACTTGGCAGGGTCC - 3’	NM_214057
***PPARG***	F: 5’ - TTAGATGACAGCGACCTGGC - 3’ R: 5’ - GTGAAGGCTCATGTCCGTCT - 3’	NM_214379
***IL1B***	F: 5’ - GTACATGGTTGCTGCCTGAA - 3’ R: 5’ - TGGCACACTCACCCCAAAG - 3’	NM_214055
***SPP1***	F: 5’ - CACATTGTAGCGAGGTGGGA - 3’ R: 5’ - GTGACGGCTTGTATTTCTTATACGG - 3’	NM_214023
***ITGAV***	F: 5’ - GACTTCACTGCTGATGGTGC - 3’ R: 5’ - ACAAAAGATGTGGATAGTAAGGTCT - 3’	NM_001083932
***NMB***	F: 5’ - TGTGATTTCTGGTTGAGTTGCC - 3’ R: 5’ - CAAGACATACAGCAGGGACG - 3’	NM_001123145
***S100A8***	F: 5’ - CCGACATGGCAAAAAGACCC- 3’ R: 5’ - ATAAAGGAGTCATGGCAAGCTA- 3’	NM_001004045

**Table 4:** The significance (*p* values) of differences in the gene expression at 9D, 12D and 16D of early pregnancy in both RT-PCR and microarray experiments.

**RT-PCR**	**FABP3**	**IL1B**	**ITGAV**	**NMB**	**OPN**	**PPARG**	**RBP4**	**S100A11**
9D vs. 12D	0.248	0.083	0.021	0.021	0.083	0.043	0.021	0.021
9D vs. 16D	0.021	0.021	0.021	0.043	0.021	0.021	0.021	0.083
12 vs. 16D	0.083	0.083	0.564	0.0021	0.083	0.149	0.149	0.021
**Microarray**	**FABP3/A_72_P424889**	**IL1B/A_72_P463511**	**ITGAV/A_72_P051521**	**NMB/A_72_P441888**	**OPN/SPP1/A_72_P145586**	**PPARG/A_72_P441339**	**RBP4/A_72_P146486**	**S100A11/A_72_P041606**
9D vs. 12D	0.574	0.00000012	0.658	0.0432	0.0000000754	0.123	0.00456	0.000144
9D vs. 16D	0.0284	0.0000105	0.0572	0.578	0.274	0.00671	0.0189	0.0217
12D vs. 16D	0.105	0.000364	0.00836	0.0639	0.00000132	0.00865	0.807	0.000144

**Table 5:** Gene expression (Mean SD, SEM values) at 9D, 12D and 16D of early pregnancy in both RT-PCR and microarray experiments.

**Stages**	**RT-PCR**	**FABP3**	**IL1B**	**ITGAV**	**NMB**	**OPN**	**PPARG**	**RBP4**	**S100A11**
9D	Mean	0.29678507	0.2862616	0.45462323	0.039134517	0.022101078	0.293498944	0.004475395	0.002163842
9D	SD	0.181874213	0.217897791	0.066866002	0.025376539	0.011917908	0.102534468	0.003005485	0.00106232
9D	SEM	0.090937107	0.108948895	0.033433001	0.01268827	0.005958954	0.051267234	0.001502743	0.00053116
12D	Mean	0.661363926	0.772976768	1.032295435	2.742343022	0.128210738	0.768243071	1.045137864	2.988849196
12D	SD	0.792157361	0.991360659	0.550797913	1.79142117	0.258761542	0.657077116	1.372147219	5.060744924
12D	SEM	0.39607868	0.49568033	0.275398957	0.895710585	0.129380771	0.328538558	0.68607361	2.530372462
16D	Mean	2.041851004	1.940761632	1.513081335	0.218522461	2.849688183	1.938257985	1.950386742	0.008986962
16D	SD	0.952351557	1.040282257	0.745442463	0.103526723	4.720728783	1.162845646	1.020400836	0.006955678
16D	SEM	0.476175779	0.520141128	0.372721232	0.051763361	2.360364391	0.581422823	0.510200418	0.003477839
**Stages**	**Microarray**	**FABP3/A_72_P424889**	**IL1B/A_72_P463511**	**ITGAV/A_72_P051521**	**NMB/A_72_P441888**	**OPN/SPP1/A_72_P145586**	**PPARG/A_72_P441339**	**RBP4/A_72_P146486**	**S100A11/A_72_P041606**
9D	Mean	2439.201	48.485	15496.08	23200.24	344.068	238.932	25301.21	9645.699
9D	SD	1008.043083	10.89272496	5371.666	63055.29	55.352831	76.57238	36255.42	2418.796989
9D	SEM	356.3970497	3.851159844	1899.171	22293.41	19.570181	27.07242	12818.23	855.1738765
12D	Mean	2906.12	192.019	16750.34	113595	61.806	307.028	114344.7	19990.117
12D	SD	1492.354766	54.20298132	3277.125	76325.14	21.970376	63.24315	26856.04	4844.695652
12D	SEM	527.6270877	19.16364783	1158.639	26985.01	7.767701	22.35983	9495.044	1712.858574
16D	Mean	4662.605	111.572	22283.82	42044.74	459.502	445.37	109625.8	5752.108
16D	SD	2097.077529	11.64962418	3102.399	54008.59	246.97822	115.78	33988.1	1899.866899
16D	SEM	741.4288708	4.118764126	1096.864	19094.92	87.319986	40.93442	12016.61	671.7043838

## Supplementary Materials

The following are available online at https://www.mdpi.com/2075-1729/10/5/68/s1, Tables S1–S18. Table S1. The principle component analysis (PCA) of endometrial microarray data based on the submitted to GEO NCBI microarray data representing 9D, 12D and 16D of early pregnancy of PLW gilts. Table S2. Identification pregnancy-specific differential expressed gene (DEG)-transcripts without cutoff p-values by comparing endometrium transcriptome at 9th day (9D) versus 12th day (12D) of early pregnancy in Polish Large-White gilts. Table S3. Identification pregnancy-specific differential expressed gene (DEG)-transcripts without cutoff p-values by comparing endometrium transcriptome at 9th day (9D) versus 16th day (16D) of early pregnancy in Polish Large-White gilts. Table S4. Identification pregnancy-specific differential expressed gene (DEG)-transcripts without cutoff P values by comparing endometrium transcriptome at 12th day (12D) versus 16th day (16D) of early pregnancy in Polish Large-White gilts. Table S5. Identification pregnancy-specific differential expressed gene (DEG)-transcripts with cutoff p-values < 0.001 by comparing endometrium transcriptome at 9th day (9D) versus 12th day (12D) of early pregnancy in Polish Large-White gilts. Table S6. Identification pregnancy-specific differential expressed gene (DEG)-transcripts with cutoff E-values < 0.06 by comparing endometrium transcriptome at 9th day (9D) versus 12th day (12D) of early pregnancy in Polish Large-White gilts. Table S7. Identification pregnancy-specific differential expressed gene (DEG)-transcripts with cutoff p-values < 0.001 by comparing endometrium transcriptome at 9th day (9D) versus 16th day (16D) of early pregnancy in Polish Large-White gilts. Table S8. Identification pregnancy-specific differential expressed gene (DEG)-transcripts with cutoff E-values < 0.06 by comparing endometrium transcriptome at 9th day (9D) versus 16th day (16D) of early pregnancy in Polish Large-White gilts. Table S9. Identification pregnancy-specific differential expressed gene (DEG)-transcripts with cutoff p-values < 0.001 by comparing endometrium transcriptome at 12th day (12D) versus 16th day (16D) of early pregnancy in Polish Large-White gilts. Table S10. Identification pregnancy-specific differential expressed gene (DEG)-transcripts with cutoff E-values < 0.06 by comparing endometrium transcriptome at 12th day (12D) versus 16th day (16D) of early pregnancy in Polish Large-White gilts. Table S11. The description of analysed ClueGO parameters by comparing endometrium transcriptome at 9D versus 12D versus 16D of early pregnancy in Polish Large-White gilts. Table S12. Identification pregnancy-specific upregulated gene networks and pathways by comparing endometrium transcriptome at 9D versus 12D of early pregnancy in Polish Large-White gilts using ClueGO. Table S13. Identification pregnancy-specific downregulated gene networks and pathways by comparing endometrium transcriptome at 9D versus 12D of early pregnancy in Polish Large-White gilts using ClueGO. Table S14. Identification pregnancy-specific upregulated gene networks and pathways by comparing endometrium transcriptome at 9D versus 16D of early pregnancy in Polish Large-White gilts using ClueGO. Table S15. Identification pregnancy-specific downregulated gene networks and pathways by comparing endometrium transcriptome at 9D versus 16D of early pregnancy in Polish Large-White gilts using ClueGO. Table S16. Identification pregnancy-specific upregulated gene networks and pathways by comparing endometrium transcriptome at 12D versus 16D of early pregnancy in Polish Large-White gilts using ClueGO. Table S17. Identification pregnancy-specific downregulated gene networks and pathways by comparing endometrium transcriptome at 12D versus 16D of early pregnancy in Polish Large-White gilts using ClueGO. Table S18. Statistical comparison of DEGs microarray vs RT-PCR experiments using Unadjusted, FDR Benjamini Hochberg adjusted, and Bonferroni adjusted test.

## Abbreviations

**DEG-transcript-probes**	**Differential Expressed Gene-transcript-probes**
9D	9th day
12D	12th day
16D	16th day
PLW	Polish large-white
FC	Fold of change
*TG*	*Thyroglobulin*
ZBTB2	*Zinc finger and BTB domain containing-2*
CKM	*Creatine kinase, M-type*
IL1B	*Interleukin-1beta*
PTPN11	*Protein tyrosine phosphatase non-receptor type 11*
*SELENOP*	*Selenoprotein P*
*CDC42BPA*	*Cell-division cycle-42 binding protein kinase alpha*
*MINPP1*	*Multiple inositol-polyphosphate phosphatase 1*
VAV3	*Guanine nucleotide exchange factor-3*
CASC4	Cancer susceptibility candidate gene 4 protein
*COBLL1*	*Cordon-bleu WH2 repeat protein like 1*
*ACAP2*	*ArfGAP with coiled-coil, ankyrin repeat and PH domains-2*
TMEM33	Transmembrane protein 33
*KLHL15*	*Kelch-like family member 15*
S100A11	S100 calcium binding protein A11
FABP3	Fatty acid binding protein 3
*PPARG*	*Peroxisome proliferator activated receptor gamma*
*ITGAV*	*Integrin subunit alpha V*
*OPN*	*Secreted phosphoprotein 1*
*NMB*	*Neuromedin B*
*RBP4*	*Retinol binding protein 4*
RT-PCR	Reverse transcriptase polymerase chain reaction
HSP values	High-scoring segment pair values
blastn	Nucleotide-nucleotide BLAST
*ALPPL2*	*Adaptor protein, phosphotyrosine interacting with PH domain and leucine zipper 2*
RANBP17	RAN binding protein 17
*NF1B*	*Nuclear factor I B*
*SPP1*	*Secreted phosphoprotein 1*
*CST6*	*Cystatin E/M*
TGF β1	*Transforming growth factor beta1*
FGF2	*Fibroblast growth factor 2*
EGF	*Epidermal growth factor*
ABPs	Actin-binding proteins
RIN	RNA integrity number
TBP	TATA-box binding protein
*GAPDH*	*Glyceraldehyde 3-phosphate dehydrogenase*
*HPRT1*	*Hypoxanthinine guanine phosphoribosyltransferase 1*
*BACT*	*β-actin*
*CYTB*	*Mitochondrially encoded cytochrome B*
*YWHAZ*	*Tyrosine 3-Monooxygenase/Tryptophan 5-Monooxygenase Activation Protein zeta*
*B2 M*	*Beta-2-microglobulin*
*SDHA*	*Succinate dehydrogenase complex, subunit A*
*HMBSA*	*Hydroxymethylbilane synthase a*
SD	Standard deviation
NCBI	National Center for Biotechnology Information
*ACTB*	*Actin beta*
*HMBS*	*hydroxymethylbilane synthase*
*FABP3*	*Fatty acid-binding protein 3*
*IL1B*	*Interleukin 1B*
*ITGAV*	*Integrin AV*
*NMB*	*Neuromedin B*
*OPN*	*Osteopontin*
*PPARG*	*Peroxisome proliferator-activated gamma receptor*
LC480	Light cycler 480
